# Detecting morphed passport photos: a training and individual differences approach

**DOI:** 10.1186/s41235-018-0113-8

**Published:** 2018-06-27

**Authors:** David J. Robertson, Andrew Mungall, Derrick G. Watson, Kimberley A. Wade, Sophie J. Nightingale, Stephen Butler

**Affiliations:** 1 School of Psychological Sciences and Health, University of Strathclyde, Glasgow, G1 1QE UK; 2 Department of Psychology, University of Warwick, Coventry, UK

**Keywords:** Face morphs, Identity fraud, Identity verification, Individual differences, Super-recogniser, Face matching, Face recognition, Passports, Biometrics

## Abstract

Our reliance on face photos for identity verification is at odds with extensive research which shows that matching pairs of unfamiliar faces is highly prone to error. This process can therefore be exploited by identity fraudsters seeking to deceive ID checkers (e.g., using a stolen passport which contains an image of a similar looking individual to deceive border control officials). In this study we build on previous work which sought to quantify the threat posed by a relatively new type of fraud: *morphed* passport photos. Participants were initially unaware of the presence of morphs in a series of face photo arrays and were simply asked to detect which images they thought had been digitally manipulated (i.e., “images that didn’t look quite right”). All participants then received basic information on morph fraud and rudimentary guidance on how to detect such images, followed by a morph detection training task (Training Group, *n* = 40), or a non-face control task (Guidance Group, *n* = 40). Participants also completed a post-guidance/training morph detection task and the Models Face Matching Test (MFMT). Our findings show that baseline morph detection rates were poor, that morph detection training significantly improved the identification of these images over and above basic guidance, and that accuracy in the mismatch condition of the MFMT correlated with morph detection ability. The results are discussed in relation to potential countermeasures for morph-based identity fraud.

**Electronic supplementary material:**

The online version of this article (10.1186/s41235-018-0113-8) contains supplementary material, which is available to authorized users.

## Significance statement

The use of fraudulent passports for identity verification represents a significant threat to national security. Modern passports contain counterfeit prevention measures (e.g., printed patterns visible only under specific artificial illumination) which make any attempts to alter or duplicate the document itself unlikely to go unnoticed. As a result, fraudsters are now known to be focusing on obtaining FOG (fraudulently obtained but genuine) passports. FOG passports are real documents which are wrongly issued to fraudulent applicants, and they arise when a confederate, who holds a genuine passport, submits a renewal application with the photo of a similar looking client. If the mismatch between the renewal image and the image held on file goes undetected, a FOG passport is issued which can be used illegally by the client individual. In a recent advancement in this approach, criminals are seeking to increase their success rate by submitting a morphed passport photo, an image of the confederate and the client which has been digitally blended together and which retains a likeness of both individuals. Border security agencies have only recently detected the use of passport morphs, and research is required to ensure that the relevant agencies and practitioners stay one step ahead of these criminal attacks. Here we use applied psychological science to quantify morph detection rates, to assess the effectiveness of a morph detection training task, and to evaluate the use of individuals who show a high aptitude on a test of unfamiliar face matching as a potential countermeasure.

## Background

Our reliance on passport photos for identity verification is critical to our border security (*see* Robertson & Burton, 2016). Advances in passport anti-counterfeit measures (e.g., the use of printed patterns visible only under specific artificial illumination) make traditional fraud attacks (e.g., creating a fake passport or the removal and replacement of the photo) unlikely to go undetected (UK HM Passport Office Report, 2013; UK National Fraud Authority Report, 2011). As a consequence, fraudsters are now attempting to obtain FOG (fraudulently obtained but genuine) passports (ITW Security Division White Paper, 2017; Middleton, 2014). FOG passports are real documents which have been mistakenly issued to fraudulent applicants. Such documents can arise through a variety of methods, as outlined below, but the most direct route occurs when a confederate, who holds a genuine passport, submits a renewal application with the photo of a similar looking fraudster. If the mismatch between the renewal image and the image held on file goes unnoticed, a FOG passport is issued which can be used illegally by the fraudster. This type of fraud exploits a weakness that results from our reliance on unfamiliar face matching for identity verification (Jenkins, White, Van Montfort, & Burton, 2011; Kemp, Towell, & Pike, 1997; White, Kemp, Jenkins, Matheson, & Burton, 2014). Extensive research has shown that detecting that two similar looking faces are in fact different people (i.e., detecting fraud) is a challenging task and one which is prone to error (*see* Bruce et al., 1999; Burton, 2013; Davis & Valentine, 2009; Robertson, Middleton, & Burton, 2015; Young & Burton, 2017). This issue is being exacerbated by recent advances in image manipulation software which allow fraudsters to easily create morphed passport photos.

**Fig. 1:**
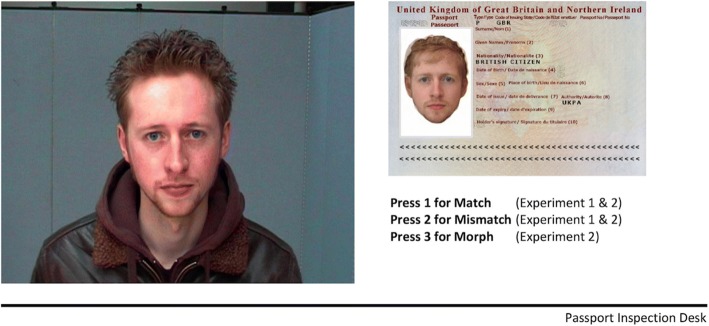
Example trial from Robertson et al. (2017). The image to the left was always known to be genuine; the task was to decide whether the passport photo was a genuine match (real photo of the same person), a mismatch (real photo of a different but similar looking person) or a morph (digitally created blend of two different face photos). (The photo in the passport frame depicts a 50% morph.)

**Fig. 2:**
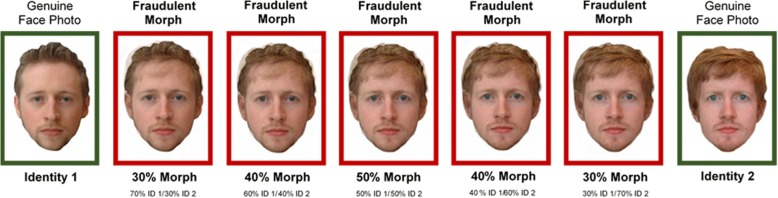
Examples of the face images used in the pre-array, post-array and morph training tasks

Internet users, and even those using smartphones, now have access to a variety of face image manipulation apps that support the digital morphing of the face photos of two different people, with such images retaining facial information that is specific to both identities (*see* Figs. 1 and 2 for examples). The advantage to the fraudster of using morphs over a similar looking confederate photo is that the image retains some of the confederate’s face and may therefore be more likely to be accepted as a match to the photo held on file at passport renewals. If the morph were to be accepted as a match, a FOG passport would be issued which could be used illegally by the fraudster for identity verification purposes (*see* Makrushin, Neubert, & Dittmann, 2017; Scherhag et al., 2017). The same process could be repeated even without access to a confederate in situations in which the fraudster has access to stolen identity details.

A recent study by Robertson, Kramer, and Burton (2017) was the first to assess the detection rates for morphed passport photos by human recognisers in a passport photo-to-face-photo matching context. As shown in Fig. 1, on each trial, participants had to decide whether to accept a passport photo as a match to a genuine face (left image), either with no prior awareness of there being morphs in the set (i.e., the applied scenario before this type of fraud came to light) or with an awareness of the morphs and the explicit instruction to detect them. The results showed that acceptance rates for 50% morphs, the grade which is most likely to confer a benefit to fraudsters, was 68% in the no-awareness condition, suggesting that these images do provide a viable route to identity fraud (*see also* Ferrara, Franco, & Maltoni, 2016). Although it is the case that morph acceptance rates fell to 21% after participants had been provided with some rudimentary morph detection guidance, 50% morphs were still a more successful form of fraud than simply using the genuine photo of another, similar looking, person.

These findings quantified morph detection rates in the presence of a photo which was known to be genuine, mirroring the morph fraud process outlined above, in which passport renewal officers compare an incoming morph with a genuine passport photo held on file. However, Robertson et al. (2017) did not address purely opportunistic morph fraud, in which a fraudster submits an entirely new passport application with a morphed photo. In this context, in which an existing passport does not exist (i.e., there is no previous comparison photo held on file), the fraudster is simply relying on the official’s failure to detect that they have submitted a digitally manipulated photo of two different people. Again, were a FOG passport to be mistakenly issued in this context, two individuals would then be able to use the same document. Despite the threat posed by this type of attack, the extent to which morphs can be detected in the absence of a comparison image that is known (or is at least very likely) to be genuine has not yet been established.

In line with the growing focus on individual differences in face recognition performance (*see* Dennett, McKone, Edwards, & Susilo, 2012; Furl, Garrido, Dolan, Driver, & Duchaine, 2011; Wilmer et al., 2010), Robertson et al. (2017) also assessed whether individual levels of accuracy on items from the Glasgow Face Matching Test (GFMT; Burton, White, & McNeill, 2010), a well-established test of unfamiliar face matching ability, predicted morph detection rates. Although recent work on ‘super-recognisers’ (SRs) has shown that SR performance tends to correlate across face-processing tasks (i.e., memory and matching; Bobak, Hancock, & Bate, 2016; Davis, Lander, Evans, & Jansari, 2016; Robertson, Noyes, Dowsett, Jenkins, & Burton, 2016), morph detection does not rely on identity recognition, and so it was not clear whether accuracy on face identification tasks would generalise to the detection of digitally manipulated face images.

Robertson et al. (2017) reported that participants’ morph detection rates did correlate with GFMT accuracy, but only for scores in the mismatch condition. That is, participants who scored highly in detecting that two similar looking faces were in fact two different people also identified a greater number of morphs. However, the full GFMT was not completed separately from the morph task, with match/mismatch accuracy being calculated from the small number of genuine match/mismatch trials used in the morph task (i.e., non-morph pairs; 7 per match/mismatch condition per participant). Therefore, although the correlation between morph detection and GFMT mismatch accuracy appears to extend recent work on the generalisability of the skills of high-performing face recognisers, or SRs, and their potential usefulness as a morph fraud counter-measure, further investigation is required to assess the reliability of this relationship.

The study by Robertson et al. (2017) provided important data in terms of quantifying the acceptance of morphed passport photos in the presence of a genuine comparison photo. In the present study we build on this work in three important ways. First, we examine morph detection rates when participants are simply asked to detect images that look as though they have been digitally manipulated, with no awareness that there are morphs in the set. This process mirrors the task facing passport-issuing officials who may be unaware of this type of fraud when confronted by a fresh application that contains a morph. Second, we contrast the effectiveness of the rudimentary morph detection guidance provided by Robertson et al. (2017), with a morph detection training task which provides participants with feedback on their performance. Third, we seek to establish the reliability of the association between mismatch accuracy on an unfamiliar face matching test and morph detection ability.

## Methods

### Participants

Eighty participants were recruited for this study during a public engagement workshop and were randomly assigned to the Training Group or the Guidance Group. Sample characteristics are reported in Table 1. There was no group difference in age (*t* < 1). The ethics committee of the School of Psychological Sciences and Health (University of Strathclyde) approved this research. All participants provided written informed consent.

**Table 1:** Sample characteristics

	Guidance Group (*n* = 40)			Training Group (*n* = 40)		
Age (years)	Mean = 30	SD = 12	Range = 16–54	Mean = 31	SD = 12	Range = 16–57
Sex (*n*)	15 Male	25 Female	63% Female	17 Male	23 Female	58% Female

### Stimuli and apparatus

#### Face images

The face morphs used in this study were selected from the set of 49 identity pairs used by Robertson et al. (2017). For each identity pair, morphs were available in 10% gradations between Identity 1 and Identity 2. However, as shown in Fig. 2, we selected only 30%, 40% and 50% morphs for use in the present study. These morphs were selected because they are within the range that is likely to confer the greatest benefit to an identity fraudster in a passport fraud context (*see* Robertson et al., 2017, for related discussion) and are therefore most likely to be presented to passport officials. Within each of the 49 identity pairs, there are two 30% morphs (i.e., 30% of Identity 1 and 70% of Identity 2; 30% of Identity 2 and 70% of Identity 1), two 40% morphs (i.e., 40% of Identity 1 and 60% of Identity 2; 40% of Identity 2 and 60% of Identity 1) and one 50% morph. Therefore, the present morph set consisted of 98 30% morphs, 98 40% morphs and 49 50% morphs. The 2 genuine images from each pair (98 images) were selected as foils. All images matched the size of real UK passport photos (3.5 cm × 4.5 cm) and were presented in colour on a white background.

#### Models Face Matching Test

Our version of the Models Face Matching Test (MFMT) consisted of 60 pairs (2 shortened versions combined) of unconstrained, highly variable face photos of male models. Within the test, as is the case with the GFMT, there are two subtests consisting of match trials (same person, different image) and mismatch trials (different, but similar looking, people) (*see* Dowsett & Burton, 2015, for further information). The MFMT is designed to be more difficult than the GFMT (Burton et al., 2010), on which it is based; it is therefore likely to provide a more sensitive measure of individual differences in face-matching ability. In addition, because the morph images were derived from GFMT faces, the use of a separate matching test rules out a potential confound in terms of the repetition of faces.

### Procedure

**Fig. 3:**
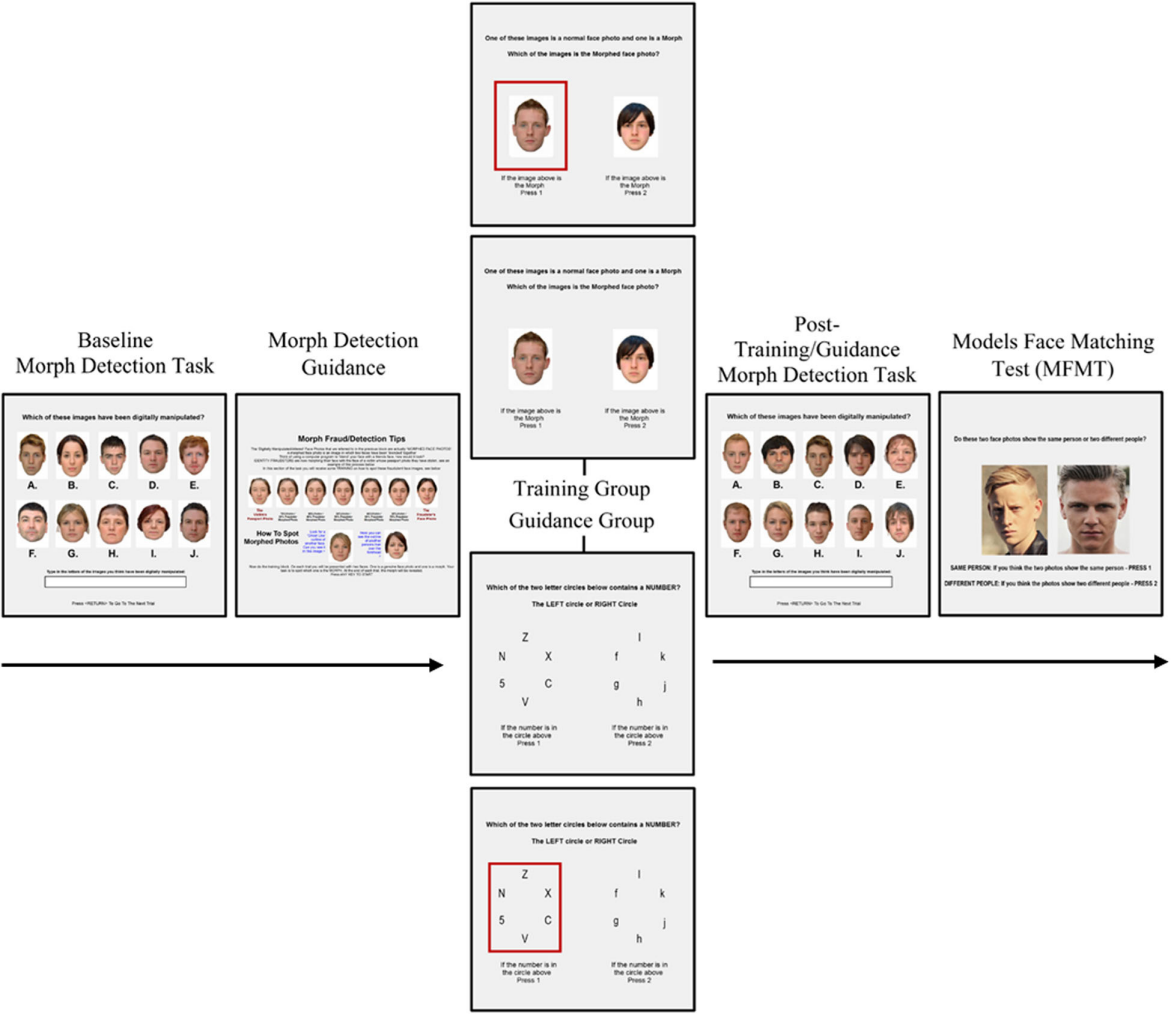
The experimental procedure. Both groups completed the baseline morph detection task and then received basic morph detection guidance. The Training Group then completed the morph training/performance feedback task, and the Guidance Group completed a selective attention control task. Both groups completed the post-guidance/training morph detection task and the Models Face Matching Test (MFMT). (Owing to copyright restrictions, images from the MFMT are not displayed in this figure; however, the images shown are a close approximation of the stimuli used and are not bound by copyright restrictions [CC0].)

The procedure for both the Guidance Group and the Training Group is summarised in Fig. 3. On entering the testing space, participants were invited to sit at a testing station, to read all of the on-screen instructions, and to begin the task when ready. All participants completed an initial morph detection task (to quantify baseline performance); they then received basic morph awareness/detection information (as used by Robertson et al., 2017), followed by a morph training task (Training Group) or a non-face (selective attention) control task (Guidance Group). The training blocks were followed by a post-guidance/training morph detection task and completion of the MFMT.

In the baseline morph detection task, participants were asked to take on the role of a passport fraud officer who is trying to detect passport photos that have been digitally manipulated. These instructions were kept deliberately vague, with no mention of morph fraud or the use of morphed passport photos in the set, to assess the extent to which individuals can generally detect digitally manipulated face photos. On each trial, participants were presented with an array containing ten face photos (five genuine, five morph; two 30%, two 40%, and one 50%). Each image was tagged with a letter (A–J), and participants were required to enter the letters, via keyboard key, for the images they thought had been digitally manipulated. There were six self-paced trials providing a total of 30 morphs and 30 genuine foils. Genuine images and morphs appeared in each image location within the array with equal probability across the task.

In Robertson et al. (2017), participants were presented with an information screen which made them aware of the use of morphs in identity fraud and in the experiment stimulus set, in addition to some basic guidance on how to detect morphs (i.e., look for a ‘ghost-like’ outline of another face; look for the outline of another person’s hair over the forehead). In the present study, participants in both groups received this basic morph detection information following completion of the baseline task.

In the morph training section of the experiment, the Training Group were then presented with 20 pairs of face photos and were made aware that one image would always be genuine and one image would always be a morph (eight 30%, eight 40%, four 50%). Participants had to detect which of the images was a morph, and as shown in Fig. 3, feedback was provided on each trial, as was additional time (3000 ms) to inspect the morph once it had been revealed. The left/right presentation of the morph was counterbalanced. The Guidance Group completed a 20-trial selective attention task in which they had to detect a number target in one of two circular letter search arrays. Response feedback was provided, and the timing within the block was identical to the morph training task. One of the letter circles was always presented in lower case, the other in upper case (left/right counterbalanced), to emulate the genuine/morph distinction experienced by the Training Group (i.e., same stimulus category, different visual properties). Both the morph training task and the selective attention task required visual search within and between left/right stimuli.

Following detection guidance or morph training, all participants completed a final block of the multi-image morph detection task, with the knowledge that the digitally manipulated images they should try to detect were morphs. The procedure for this block was otherwise identical to the baseline morph detection task. Within participants, no genuine image or morph was repeated across the experiment, and across participants, both types of face photo were selected at random for use in the baseline and post-guidance/training morph detection tasks, as well as in the morph training block. In the Training Group, 16 images were randomly selected from each set (6 for the baseline detection task, 4 for the training task, and 6 for the post-guidance/training detection task) and 12 images from each set were randomly selected for the Control Group (6/6 per baseline and post-guidance/training detection task). The testing session ended upon completion of the MFMT.

## Results

### Baseline morph detection performance

As shown in Table 2, the mean detection sensitivity score (*d'*) for each grade of morph was poor, with overall percentage hit rates at chance level (48%). Importantly, 2 × 3 mixed design analysis of variance (ANOVA) on *d'* and criterion *c* scores, with the factors of group (Training, Guidance) and morph grade (30%, 40%, 50%), revealed no group differences on either measure (*p* > .116). The analysis also revealed a bias towards the detection of 50% and 30% morphs over 40% morphs [*F*(2, 156) = 10.37, *p* < .001, η_p_^2^ = .12 for the main effect of morph grade on *c* scores; *p* < .005 for the morph grade differences]. The lack of group differences in the baseline morph detection task confirms that any post-training effects cannot be the result of baseline differences in detection performance.

**Table 2:** Mean performance scores both pre- and post-guidance/training

	Baseline Performance							
	Guidance Group				Training Group			
	Hits (%)	FA (%)	*d'*	*c*	Hits (%)	FA (%)	*d'*	*c*
30% Morphs	53	23	0.76	0.36	46	27	0.47	0.38
40% Morphs	39	23	0.38	0.55	40	27	0.31	0.46
50% Morphs	59	23	0.96	0.26	52	27	0.56	0.34
All Morphs	50	23	0.70	0.39	45	27	0.45	0.40
	Post-Guidance/Training Performance							
	Guidance Group				Training Group			
	Hits (%)	FA (%)	*d'*	*c*	Hits (%)	FA (%)	*d'*	*c*
30% Morphs	65	7	2.15	0.60	74	7	2.48	0.50
40% Morphs	73	7	2.41	0.47	79	7	2.69	0.39
50% Morphs	75	7	2.32	0.52	85	7	2.69	0.39
All Morphs	71	7	2.29	0.53	79	7	2.62	0.43

*Abbreviations: d′* Detection sensitivity, *c* Criterion score, *FA* False alarm

*Note*: Because of the experimental design, when assessing morph detection sensitivity at each morph grade, the FA rate remains constant as the different morph grades were presented within the same array

All Morphs show performance averaged across morph grades (30%, 40%, 50%)

### Post-guidance/training morph detection performance

Participants’ mean detection sensitivity (*d'*) scores were entered into a 2 × 2 × 3 mixed design ANOVA with the between-subjects factor of group (Training Group, Guidance Group) and the within-subjects factors of time (Before Guidance/Training, After Guidance/Training) and morph grade (30%, 40%, 50%). The descriptive statistics are presented in Table 2.

The ANOVA revealed main effects of morph grade and time, which were qualified by a morph grade × time interaction [*F*(2, 156) = 11.06, *p* < .001, η_p_^2^ = .12]. As expected, morph detection rates were significantly higher in the post-guidance/training morph detection task at each morph grade than in baseline performance (*p’s* < .001), and the source of the interaction was a greater improvement in performance for 40% morphs (*M* = 2.20, *SD* = 1.30) than in the other two morph grades (*M* = 1.70, *SD* = 1.23 for 30% morphs; *M* = 1.74, *SD* = 1.41 for 50% morphs; *p* < .001 for the differences). This is likely to be a result of the initial lower baseline detection rates for the 40% morphs reported above. These levels of improvement led to greater detection sensitivity for 40% (*M* = 2.55, *SD* = 1.21) and 50% (*M* = 2.50, *SD* = 1.15) morphs than for 30% morphs (*M* = 2.31, *SD* = 1.09; *p’s* < .026 for the differences) in the post-guidance/training morph task. This pattern of detection rates replicates the findings of Robertson et al. (2017; Experiment 2) in showing that morphs get easier to detect the closer they get to the 50% ratio (of Identity 1 and Identity 2), when participants are made aware of morph fraud and their presence in the image set.

**Fig. 4:**
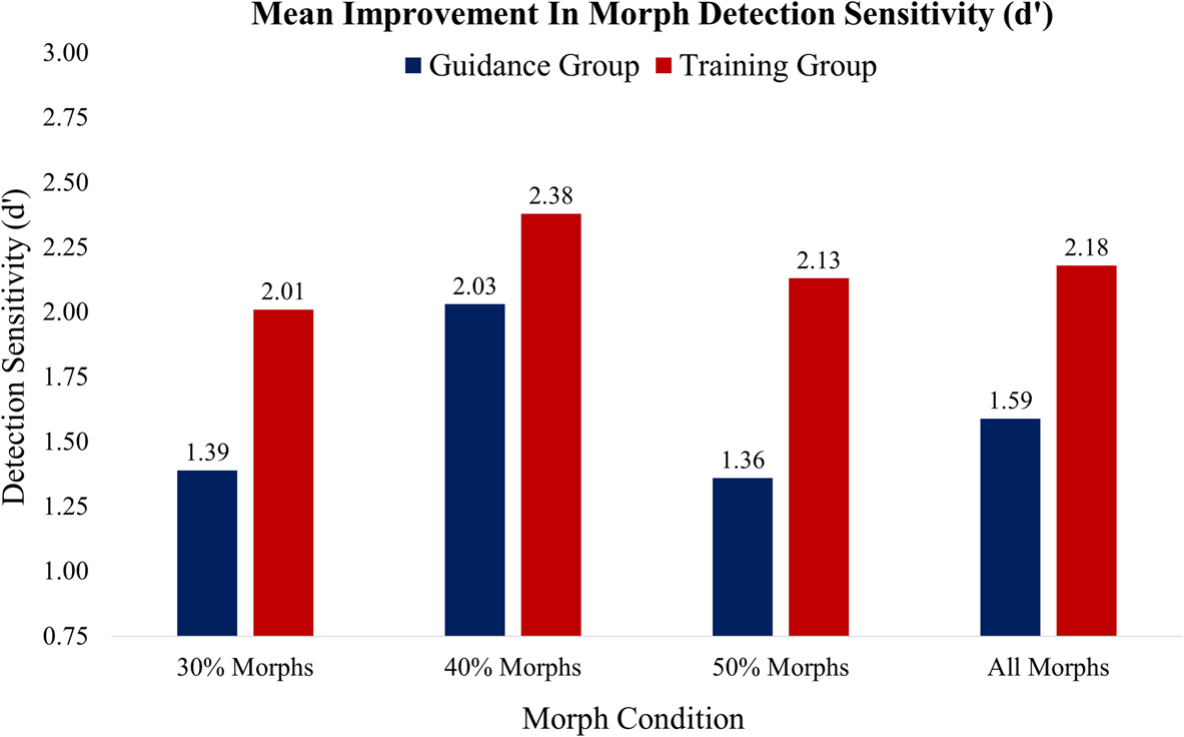
Mean detection sensitivity scores (*d'*) showing the improvement in morph detection performance between the pre- and post-training arrays. The data are presented as a function of morph grade and group

Although there was no overall main effect of group (*F* < 1), there was a significant interaction between group and time [*F*(1, 78) = 5.36, *p* = .023, η_p_^2^ = .06]. While detection sensitivity significantly increased in the post-guidance/training arrays in both groups [*t*(39) = − 10.36, *p* < .001 for the Guidance Group; *t*(39) = − 10.82, *p* < .001 for the Training Group], importantly, as shown in Fig. 4, the source of the interaction was a significantly greater increase in morph detection sensitivity for the Training Group (*M* = 2.18, *SD* = 1.27) compared to the Guidance Group [*M* = 1.59, *SD* = 0.97; *t*(78) = 2.31, *p* = .023, *d* = .522 for the difference]. The remaining two-way interaction and the three-way interaction were not significant (*p’s* > .215), indicating that the improvement in morph detection performance as a result of the morph training task was equally effective at each morph grade. A final ANOVA on post-guidance/training array criterion *c* scores, with the factors of group, morph grade and time, revealed no significant group differences (all *F’s* < 1).

These findings show that our rudimentary detection guidance plus training led to a greater improvement in morph detection rates than the rudimentary detection guidance alone. In overall percentage hit terms, as shown in Table 2, the basic morph awareness information received by the Guidance Group led to a 21% increase in morph detection rates to 70%, whereas in the Training Group the improvement was 34%, leading to a 79% overall morph hit rate.

#### Morph training and control task performance

For the Training Group, mean accuracy on the morph training task was 89% (*SD* = 15%, range = 35%–100%), showing that even when participants were made aware that one of two images was definitely a morph, perfect levels of performance were not achieved. For the Guidance Group, mean accuracy on the selective attention task was 98% (*SD* = 3%, range = 90%–100%).

### Individual differences in morph detection improvement

**Fig. 5:**
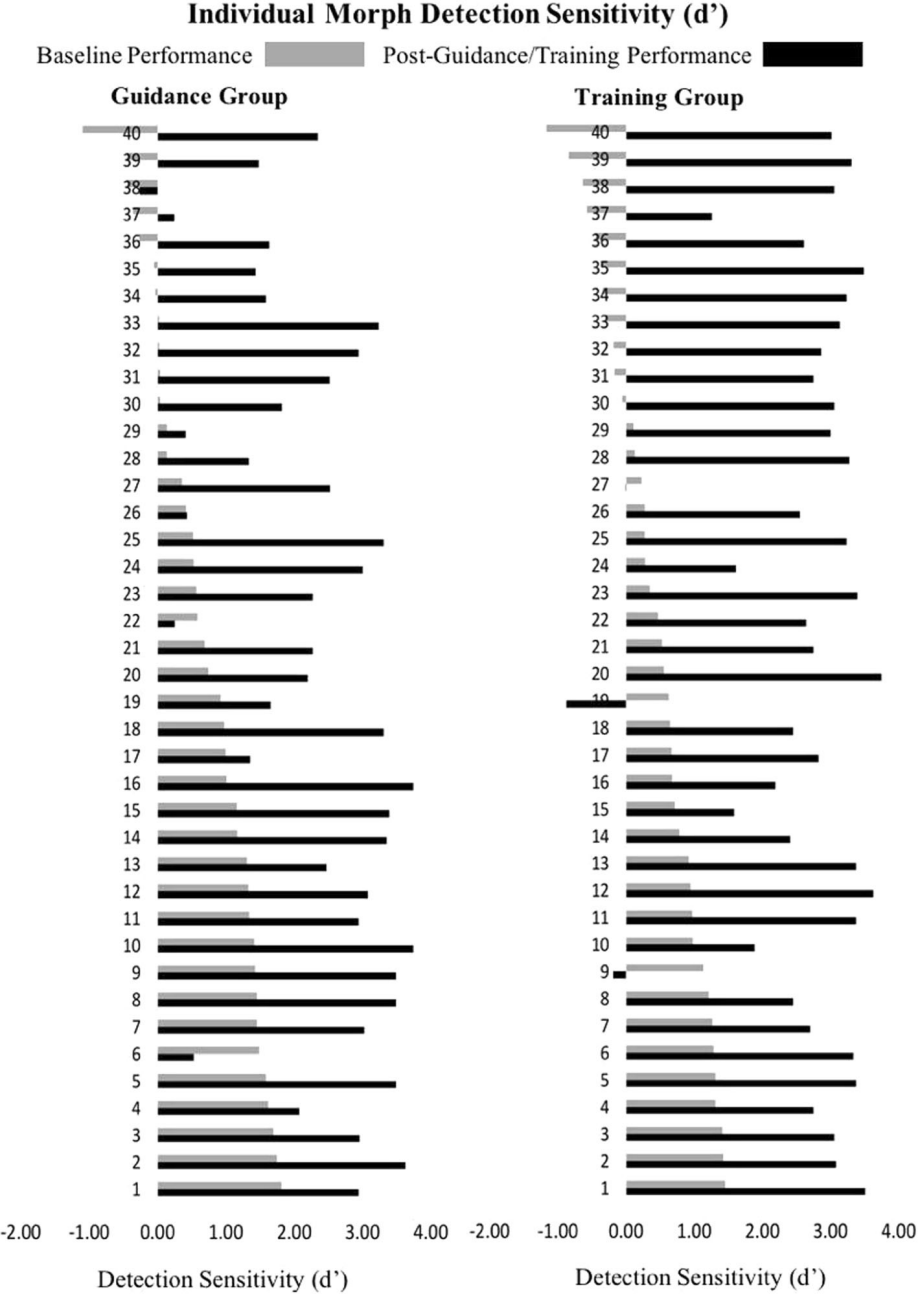
Individual *d'* scores for each participant in each group in the baseline morph detection task (*grey*) and the post-guidance/training morph detection task *(black)*. Within each group (Guidance, Training), participants’ scores are arranged from high to low performers (1–40) in the pre-guidance/training morph detection task

The findings described above, reported at the group level, show that targeted morph training enhanced detection rates to a greater extent than basic morph detection guidance; yet, it remains unknown whether the training benefits all participants equally. To assess this, we split each group into two sections, each containing the 20 participants with the lowest and highest *d'* scores (averaged across the conditions) from the baseline detection task. We then calculated each participant’s improvement in detection sensitivity by subtracting their *d'* score post-guidance/training from their baseline score. Although, as described above, initial performance on the baseline detection task was poor at the group level, there was still a degree of variation in scores, as shown in Fig. 5. Baseline morph detection scores were significantly different between the low and high performers in each group (*p* < .001).

The analysis revealed that, for low performers, the mean improvement in detection sensitivity scores was significantly higher for those in the Training Group (*M* = 2.83, *SD* = 1.04) than for those in the Guidance Group [*M* = 1.67, *SD* = 1.08; *t*(38) = 3.465, *p* = .001]. However, no such difference was found between the groups for high performers (*M* = 1.52, *SD* = 1.15 for Training; *M* = 1.51, *SD* = .86 for Guidance; *t* < 1). This suggests that the targeted morph training task is likely to be effective for individuals who initially are particularly poor at detecting these images, whereas such training is not likely to facilitate further improvement in high performers over and above rudimentary morph detection guidance.

### Individual differences in morph detection and MFMT accuracy

There were no group differences in MFMT scores, either for overall accuracy or for the individual match and mismatch conditions (*F* < 1). Across the groups, mean MFMT accuracy was 73% (*SD* = 10%, range = 40%–97%), a score that is in line with previously published norms for this test (*see* Dowsett & Burton, 2015; Robertson et al., 2016). Previous work by Megreya and Burton (2007) has shown that individuals’ accuracy on the match and mismatch components of unfamiliar face matching tests do not correlate. That is, one can perform well at detecting that two unfamiliar faces show the same person and poorly at detecting when they are different people, and this pattern was replicated in the current data (*r* = −.178, *n* = 80, *p* = .113).

#### Baseline morph detection performance

For both the Guidance Group and the Training Group, there were no significant correlations between baseline *d'* scores and MFMT overall accuracy (*r* = .066, *n* = 40, *p* = .685 for Guidance; *r* = −.027, *n* = 40, *p* = .868 for Training), match trial accuracy (*r* = .010, *n* = 40, *p* = .951 for Guidance; *r* = −.184, *n* = 40, *p* = .256 for Training), or mismatch trial accuracy (*r* = .072, *n* = 40, *p* = .657 for Guidance; *r* = .137, *n* = 40, *p* = .398 for Training). This indicates that when individuals were given only very general task instructions (pick out images that have been digitally manipulated), high levels of accuracy on a face identification task do not confer a morph detection advantage. This could be due to the fact that detection rates are consistently poor across participants.

#### Post-guidance/training morph detection performance

For the Guidance Group, there were no correlations between morph detection sensitivity scores in the post-guidance/training arrays and overall MFMT accuracy (*r* = .124, *n* = 40, *p* = .448) or match trials accuracy (*r* = −.210, *n* = 40, *p* = .194). However, there was a significant positive association between participants’ MFMT mismatch accuracy and overall morph detection sensitivity (*r* = .395, *n* = 40, *p* = .012). This suggests that face identification ability does generalise to morph detection performance when specific task instructions are provided. This finding replicates the effect reported by Robertson et al. (2017) in a larger sample and with different face-matching and morph detection tasks. Although this suggests that individuals who perform highly on the mismatch conditions of unfamiliar face matching tests are likely to be better at detecting face morphs, in both the current study and in Robertson et al. (2017), the correlations have been modest in size and marginal in significance. Further research is therefore still required to confirm how robust this effect is.

For the Training Group, there were no significant correlations between *d'* scores in the post-guidance/training arrays and overall MFMT accuracy (*r* = .061, *n* = 40, *p* = .710), match trial accuracy (*r* = −.150, *n* = 40, *p* = .356) or mismatch trial accuracy (*r* = .205, *n* = 40, *p* = .205). At first glance, it is surprising that we do not replicate the relationship between mismatch trial performance on the MFMT and post-training morph detection rates found in the Guidance Group and in Robertson et al. (2017). However, this finding is likely to follow from the fact that morph training provides an advantage to low, but not high, performers, over and above detection guidance. That is, *d'* scores have increased in this group for low-performing individuals but not proportionately in high-performing individuals, which is likely to have eliminated the equivalent correlation found in the Guidance Group.

## Discussion

In this study we assessed the extent to which morphed passport photos could be detected without awareness of them in the stimulus set, and whether morph detection/performance feedback training could improve detection rates over rudimentary guidance. In addition, we assessed whether face-matching aptitude on the MFMT was associated with morph detection ability. Our findings show that overall morph hit rates, when individuals were given only a general instruction to detect digitally manipulated images (i.e., “images that don’t look quite right”), were at chance level. This finding is in line with recent work by Nightingale, Wade, and Watson (2017), who reported poor detection rates—just above chance level—for digitally manipulated scenes. That participants were initially unable to detect the morphs is a cause for concern for passport-issuing authorities, because it suggests that the opportunistic use of morphs with new passport applications is likely to go undetected when staff are unaware, or are not actively seeking to detect, this type of fraud.

For the morph detection training, performance was not perfect, with 10% errors occurring on average, despite participants’ being made aware that one of the two images would always be genuine. This shows that even when individuals are explicitly asked to detect morphs by scanning both images in detail, some morphs remain difficult to detect. Despite this, performance on the post-guidance/training arrays show the clear benefit of having completed the morph training task in comparison to rudimentary morph detection guidance received by the Guidance Group and as used previously by Robertson et al. (2017).

One of the key aspects of the morph training task is likely to be the use of performance feedback (*see* White, Kemp, Jenkins, & Burton, 2014). On each trial, after a response was made, the morph image was always revealed, and it remained on-screen for a short period of time for further inspection. This is likely to be helpful in learning to identify the cues that give rise to the detection of morphs, particularly on trials in which participants failed to detect the morph. Although our short (20 trials) targeted morph training did improve morph detection rates to around 80% of morphs detected, this still falls short of perfect levels of performance.

Moreover, morph training was effective only for individuals who performed particularly poorly on the initial baseline detection task. Morph training provided no additional detection advantage, over and above basic detection guidance, for individuals who performed relatively well at the outset of the task. White, Kemp, Jenkins, and Burton (2014) reported a similar effect in which trial-by-trial feedback training conferred the greatest advantage to low performers on the GFMT. They noted that accuracy in high performers may not have improved owing to constraints on perceptual processing or limitations to the information provided in the image. The same thought could apply to the present study, that high performers reach task ceiling performance after receiving the basic detection guidance, and limitations within the images (i.e., images with very few morph ‘cues’) or to their perceptual ability (i.e., a limit to the ability to perceptive the least obvious manipulations) prevent further improvement in performance. If that is the case, then the more sophisticated the morph, the less likely it is that a human operator would detect it, even when motivated to try.

Although the morph training task did improve performance in low performers, future research should determine whether this is a transient or lasting effect (i.e., manipulate the time interval between training and test), because it is likely that officials may not encounter this type of fraud on a high-frequency basis. In addition, further studies should assess whether training on one morph set leads to an improvement in the detection of morphs derived from a different image set, as passport officials/border control officers are likely to encounter highly variable morphs, both in terms of quality and construction. Although security is paramount, if practitioners were able to share morphs recovered from fraudsters with researchers, training methods with greater ecological validity could also be developed.

Our study also revealed a positive association between accuracy on the MFMT mismatch condition and overall morph detection ability in the Guidance Group. That is, participants who perform better at identifying that two similar looking faces are in fact different people also detect a greater proportion of morphs. This finding replicates the tentative effect reported by Robertson et al. (2017) and provides further evidence—using a larger sample, a separate face-matching test and a different morph task—that this may be a reliable effect. However, the correlation reported in the present study, like that described by Robertson et al. (2017), was modest in size and only marginally significant; therefore, further large-scale studies are required to assess how robust this relationship is. Despite this, Metropolitan Police SRs have been shown to outperform control individuals on the MFMT (Robertson et al., 2016), which leads to the intriguing possibility, should the correlation be found to be reliable, that such individuals could also perform better than the present sample at morphed passport photo detection. If that were found to be the case, such individuals could be deployed as a potential countermeasure to morph fraud (*see* Noyes, Phillips, & O’Toole, 2017, for a recent review on SR research).

Post-guidance/training morph detection rates conformed to the pattern reported by Robertson et al. (2017), with morphs closer to the 50% centre point being easiest to detect. However, in the baseline morph detection task, there was a slight bias away from categorising 40% morphs as manipulated images. It could be the case that 30% and 50% morphs were detected more often because the two face shapes have not yet blended completely (30%) and the cues related to overlapping hair, for example, from one identity are present to a greater degree (50%). However, when participants are made aware of what to look for when detecting morphs, both types of cue are then apparent, albeit to lesser degrees, in the 40% morphs, which could have led to the greatest improvement in detection sensitivity in that condition.

Although the present study focuses on the use of modern digital tools to commit sophisticated identity fraud, the use of morphs are part of the wider evolution in identity deception techniques. For example, in one case of identity fraud, it was reported that a young Asian man had stolen the passport of an elderly white man. Using a hyper-realistic silicone mask which looked like the legitimate passport owner, the individual was able to pass several identity checks at a Hong Kong airport. The use of the mask was discovered only when the perpetrator decided to remove it mid-flight (Zamost, 2010). Despite this report occurring almost a decade ago, and with other such reports also occurring (*see* Bernstein, 2010), a recent study by Sanders et al. (2017) provided the first assessment of participants’ ability to detect the use of a hyper-realistic silicone mask as a method of physical identity deception. Their research showed that only 6% of participants detected that a confederate was wearing a mask at spontaneous or prompted report, and this rose to just 57% when asked directly. These findings show that hyper-realistic masks do provide a viable route to identity fraud, and taken together with the findings of the present study, they show that a concerted approach is required to ensure that we focus both on combatting advances in digital and physical identity fraud techniques.

## Conclusions

Providing passport-issuing officials with morph detection training is likely to significantly decrease the likelihood of FOG passports’ being issued with a morphed photo. Our findings suggest that individuals who score highly in detecting that two similar looking faces are in fact different people may also be better at detecting morphs. Further research is required to assess whether SRs could be deployed as an effective morph fraud countermeasure, and a combined effort is required to combat both digital (morphs) and physical (hyper-realistic masks) advances in identity deception techniques.

## Additional file

Additional file 1: Summary Data. (XLSX 32 kb)

## Abbreviations

GFMT: Glasgow Face Matching Test
MFMT: Models Face Matching Test
SR: Super-recogniser
